# Supplementary figures and images for: Prostate Cancer in Renal Transplant Recipients: Results from a Large Contemporary Cohort
Source: Cancers (Basel). 2022 Dec 28;15(1):189. doi: 10.3390/cancers15010189 (PMC9818510; doi:10.3390/cancers15010189)

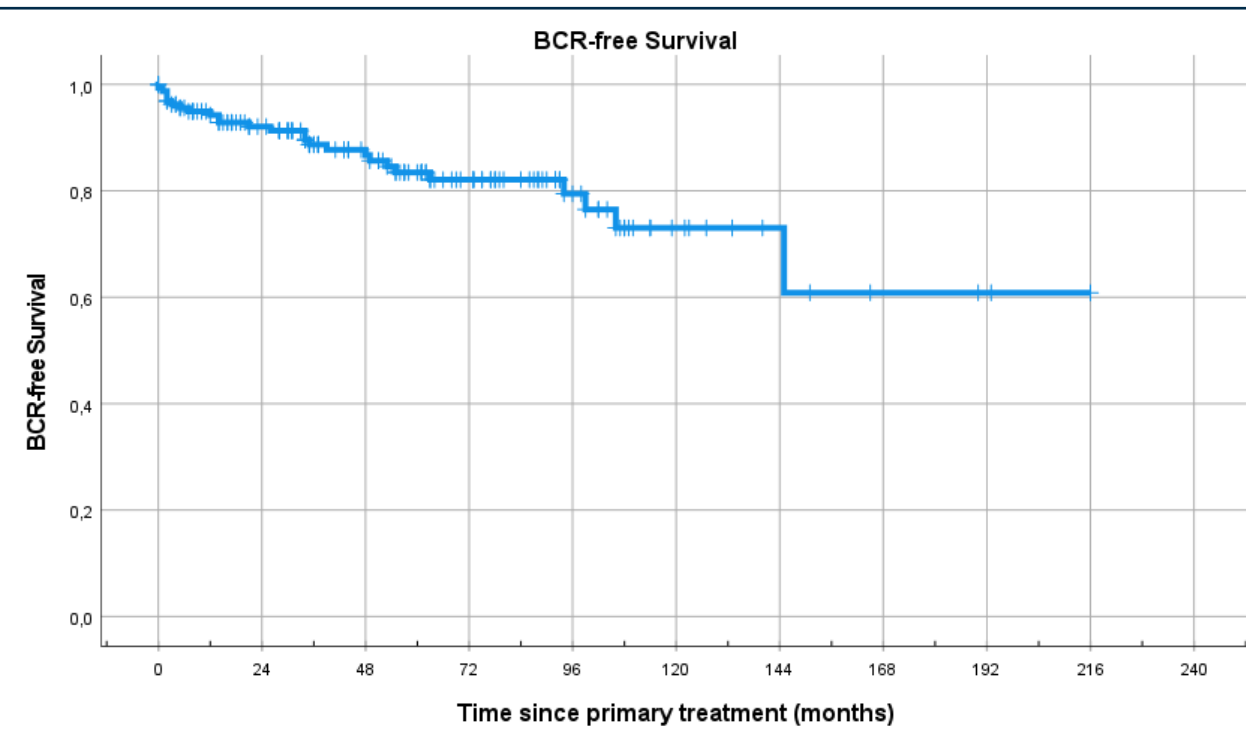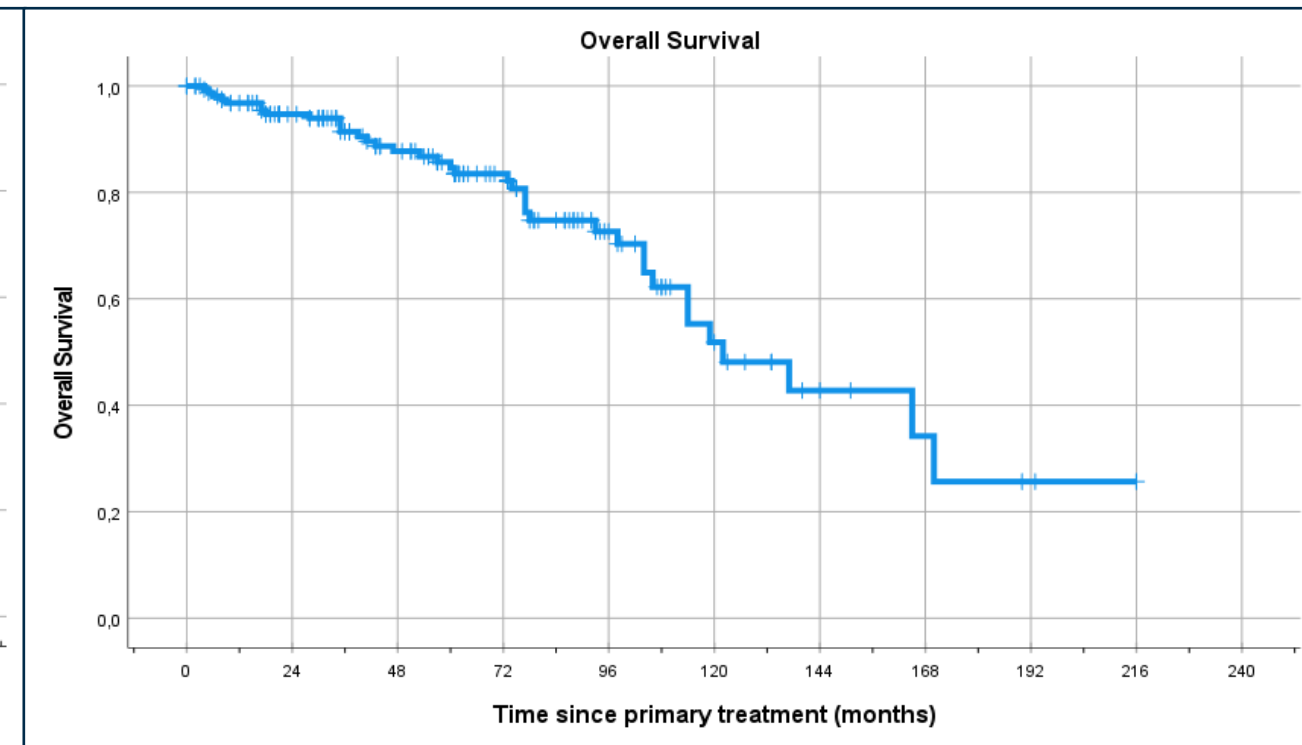

Supplement: Supplementary file 1 [file cancers-15-00189-s001.zip › Supplementary Figure S1.pdf]
